# Diagnosis and Management of a Patient With Chronic Lymphocytic Leukemia and a Concurrent Plasmacytoma

**DOI:** 10.1155/2024/8870681

**Published:** 2024-09-24

**Authors:** Danielle C. Thor, Rohan Umrani, Jack Bergal, Charles Yang, Richard Gordon

**Affiliations:** Department of Internal Medicine Thomas Jefferson University, Philadelphia, Pennsylvania, USA

**Keywords:** chronic lymphocytic leukemia, CLL, hematology, lymphoma, oncology, plasmacytoma

## Abstract

Chronic lymphocytic leukemia (CLL) typically presents as an indolent disease with a benign disposition in most patients. In select patients, CLL can progress into a more aggressive disease via its original morphology, following a Richter transformation to an alternative non-Hodgkin's lymphoma, or with the concomitant development of multiple myeloma. In an extremely rare subset of individuals with CLL, an extramedullary plasmacytoma may coexist. This case report seeks to describe the diagnosis and treatment of a patient with concurrent CLL and a plasmacytoma.

## 1. Introduction

Of the breadth of hematological malignancies, chronic lymphocytic leukemia (CLL) is viewed as a common lymphoproliferative disorder in Western populations [1]. Most patients with CLL tend to follow an indolent disease course, with an approximate 5-year relative survival rate of 88.0% [2]. However, progressive involvement of disease remains a concern and may necessitate further diagnostic workup and treatment. Causes of disease progression in CLL can be stratified into progression of the original pathology, a pathological transformation as seen in Richter syndrome, or the appearance of a concomitant hematological malignancy [3].

In the setting of a secondary malignancy, CLL progression can parallel the development of hematological malignancies not associated with a Richter transformation, myeloproliferative disorders, or select solid tumors [4]. In an exceedingly rare subset of individuals, the presence of an extramedullary plasmacytoma may coexist with active CLL disease. A scarcity of such cases exists in the scientific literature, thereby necessitating further identification and review of this uncommon patient population. This case report details the diagnosis and treatment of a patient found to have both CLL and a concurrent plasmacytoma at the time of diagnosis.

## 2. Case Presentation

A 59-year-old male with a past medical history of ADHD and bilateral hearing loss secondary to occupational exposures presented to his local emergency department with a complaint of right-sided mouth pain. An initial computed tomography (CT) of the facial bones was only revealing for dental disease of the left second molar with early periapical disease. The patient's complaints were thereby assessed as secondary to a dental infection and was originally discharged with a course of oral clindamycin. He completed the antibiotic course as prescribed but experienced no relief of his symptoms and returned to the emergency department for reevaluation and admission thereafter. Of note, his family history consisted of his father passing from diffuse large B cell lymphoma (DLBCL), his paternal uncle passing from anaplastic thyroid cancer, and his brother being diagnosed with an unknown kidney malignancy at the time of service.

On admission, the pain progressed despite more aggressive antibiotic and analgesic treatment and was paralleled with new complaints of facial numbness and dysgeusia. Outpatient magnetic resonance imaging (MRI) of the brain was obtained and demonstrated several enhancing lesions, including an extramedullary mass at the right side of the skull base. Further CT imaging of the chest, abdomen, and pelvis demonstrated additional numerous, indeterminate scattered lytic lesions alongside the aforementioned skull base mass. Laboratory workup was notable for hypercalcemia, a kappa free light chain of 73 mg/L (normal 3.30–19.40 mg/L), a kappa/lambda ratio of 9.2 (normal 0.26–1.65), and a 1.7 g M-sike with IgG-K banding on serum protein electrophoresis (SPEP). Several complete blood counts, including white blood cell counts, hemoglobin, and platelet counts were all within normal limits throughout his diagnostic workup.

**Figure 1:**
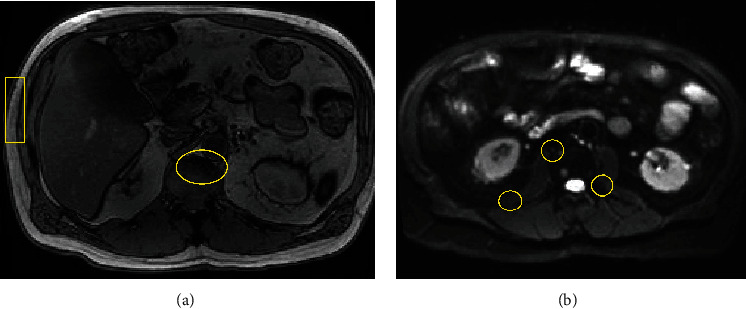
Key images from the MRI of the abdomen and pelvis obtained which demonstrate several of the over 40 abnormal foci of marrow throughout the skeletal architecture. These images highlight lesions visualized within the ribs and vertebrae (a) and within the bilateral iliac crests (b). (Of note, the majority of the patient's imaging was completed at an outpatient radiology center, and only imaging reports were available at the time of this report).

A final MRI of the abdomen and pelvis was obtained and ultimately demonstrated over 40 abnormal foci of marrow identified within the ribs, lower thoracic spine, upper lumbar spine, and iliac bones Figure 1. An additional positron-emission tomography/computed tomography (PET/CT) was obtained, which further delineated the presence of multiple intensely hypermetabolic bone marrow lesions and an additional hypermetabolic extramedullary mass at the skull base.

A biopsy of the basal skull mass was obtained which confirmed the presence of a plasmacytoma. Fluorescent in-situ hybridization (FISH) analysis of the biopsied tissue demonstrated gain of ATM, TP53, and NF1 mutations. An additional FISH analysis of a peripheral blood sample failed to demonstrate any evidence of trisomy 12, P53 deletion or amplification, D13S319, or ATM deletion. Furthermore, three separate bone marrow biopsies demonstrated CD5+ and CD23+ B-cell lymphoma consistent with CLL and/or small lymphocytic leukemia (SLL), as well as a clonal plasma cell neoplasm. On further analysis, the aforementioned bone marrow biopsies also demonstrated a 13q14 deletion, which was not originally seen on the skull biopsy results. This distinct deletion confirmed that the plasmacytoma and CLL were separate malignancies not originating from the same clone.

Given the patient's burden of radiological disease, the decision was made between a multidisciplinary team to pursue active treatment via combination therapy. His CLL regimen consisted of obinutuzumab and venetoclax for 6 cycles followed by a year of venetoclax maintenance therapy. His plasmacytoma was managed with two courses of gamma radiation therapy. At the time of this report, the patient has completed his maintenance therapy and maintained a complete metabolic response with no active disease noted on repeat imaging obtained 1 year and 2 years following the initiation of treatment.

## 3. Discussion

The development of a concomitant extramedullary plasmacytoma in the setting of CLL remains both a theoretical rarity and a scarcely reported phenomena in the scientific literature [5–7]. In addition, a select few reports detail the transformation of CLL to a plasmacytoma, but not their combined appearance in the same diagnostic workup with different clonal origins [8, 9]. Amongst these cases, most noted the development of the plasmacytoma several years after the original discovery of CLL, thereby further highlighting the novelty of this case.

The variety of diagnostic techniques available in the oncological setting, such as FISH analysis, kappa/lambda chain analysis, and SPEP, allow for precise determination of different origins to each malignancy. When presented with the unique situation of differentiating between the origins of CLL and a concurrent plasmacytoma, it is reasonable to assume FISH analysis is the optimal etiological methodology at this time. Moving forward, next-generation DNA sequencing and/or “Tumor ID” assays from liquid or solid biopsies may provide additional diagnostic utility in the setting of either hematological malignancy [10]. However, further validation of such testing remains necessary before it can be reasonably applied in these scenarios, as discussed in management of similar plasma cell dyscrasias [11].

The treatment of these combined malignancies requires a tailored therapeutic approach. Extramedullary plasmacytomas can be curatively treated with surgery and/or radiation depending on lesions size, location, lymph node involvement, and generalized risk stratification [12]. Therefore, it is reasonable to pursue both surgical oncology and/or radiation oncology treatment for the plasmacytoma while simultaneously designing an additional treatment plan for the concomitant CLL. Management of CLL may follow its typical indolent course, but additional surveillance should be considered for possible accelerated disease progression and/or Richter's transformation in the setting of both a secondary malignancy and dueling therapeutic approaches.

Although this case relies upon a single-patient report, its unique presentation of comparable, competing hematological malignancies enhances its significance as an advisory report for clinicians. Continued investigation of the incidence of these concurrent malignancies and optimal treatment strategies is warranted. Furthermore, the case presented includes an extensive family history of aggressive malignancies, including a paternal primary DLBCL. A greater study of any potential hereditary syndromes in these associated patient populations may be warranted to improve prognostication and disease management.

## 4. Conclusion

This case report details the diagnostic and treatment approach taken for a patient with CLL and a concurrent extramedullary plasmacytoma. In doing so, further attention is provided to this rare potential complication of an otherwise common hematological malignancy.

## References

[1] Siegel R. L., Miller K. D., Jemal A. (2020). Cancer statistics, 2020. *CA: A Cancer Journal for Clinicians*.

[2] Division of cancer control and population statistics (2020). *Cancer stat facts: leukemia-chronic lymphocytic leukemia (CLL)*.

[3] Mukkamalla S. K. R., Taneja A., Malipeddi D., Master S. R. (2023). Chronic lymphocytic Leukemia. *StatPearls*.

[4] Tadeusz R. (2004). Second malignancies and Richter's syndrome in patients with chronic lymphocytic leukemia. *Hematology*.

[5] Arias-Santiago S., Aneiros-Fernandez J., Giron-Prieto M. S., Arrabal-Polo M. A. (2010). Primary plasmacytoma of the penis associated with chronic lymphocytic leukemia. *Journal of the American Academy of Dermatology*.

[6] Chantepie S. P., Cabrera Q., Mear J. B., Salaun V., Lechapt-Zalcman E., Macro M. (2015). Unusual extramedullary plasmacytoma: a rare but possible cause of lymphadenopathy in chronic lymphocytic leukemia. *Case Reports in Medicine*.

[7] Yahata N., Iwase O., Iwama H. (2000). Chronic lymphocytic leukemia complicated by plasmacytoma originating from different clones. *Leukemia & Lymphoma*.

[8] Aslaner M., Aslan C., Ergen S., Tekin I., Ertop I. (2014). PP-044 a rare case of plasmacytoma followed chronic lymphocytic leukemia. *Leukemia Research*.

[9] Pines A., Ben-Bassat I., Selzer G., Ramot B. (1984). Transformation of chronic lymphocytic leukemia to plasmacytoma. *Cancer*.

[10] Rodríguez-Vicente A. E., Bikos V., Hernández-Sánchez M., Malcikova J., Hernández-Rivas J. M., Pospisilova S. (2017). Next-generation sequencing in chronic lymphocytic leukemia: recent findings and new horizons. *Oncotarget*.

[11] Bolli N., Genuardi E., Ziccheddu B., Martello M., Oliva S., Terragna C. (2020). Next-generation sequencing for clinical management of multiple myeloma: ready for prime time?. *Frontiers in Oncology*.

[12] Alexiou C., Kau R. J., Dietzfelbinger H. (1999). Extramedullary plasmacytoma: Tumor occurrence and therapeutic concepts. *Cancer*.

[13] Thor D. C., Umrani R., Bergal J., Yang C., Gordon R. Diagnosis and management of a patient with chronic lymphocytic leukemia and a concurrent plasmacytoma: an overview of a case report.

